# Adding a Little Reality to Building Ontologies for Biology

**DOI:** 10.1371/journal.pone.0012258

**Published:** 2010-09-03

**Authors:** Phillip Lord, Robert Stevens

**Affiliations:** 1 School of Computing Science, Newcastle University, Newcastle-upon-Tyne, United Kingdom; 2 School of Computer Science, The University of Manchester, Manchester, United Kingdom; Miami University, United States of America

## Abstract

**Background:**

Many areas of biology are open to mathematical and computational modelling. The application of discrete, logical formalisms defines the field of biomedical ontologies. Ontologies have been put to many uses in bioinformatics. The most widespread is for description of entities about which data have been collected, allowing integration and analysis across multiple resources. There are now over 60 ontologies in active use, increasingly developed as large, international collaborations. There are, however, many opinions on how ontologies should be authored; that is, what is appropriate for representation. Recently, a common opinion has been the “realist” approach that places restrictions upon the style of modelling considered to be appropriate.

**Methodology/Principal Findings:**

Here, we use a number of case studies for describing the results of biological experiments. We investigate the ways in which these could be represented using both realist and non-realist approaches; we consider the limitations and advantages of each of these models.

**Conclusions/Significance:**

From our analysis, we conclude that while realist principles may enable straight-forward modelling for some topics, there are crucial aspects of science and the phenomena it studies that do not fit into this approach; realism appears to be over-simplistic which, perversely, results in overly complex ontological models. We suggest that it is impossible to avoid compromise in modelling ontology; a clearer understanding of these compromises will better enable appropriate modelling, fulfilling the many needs for discrete mathematical models within computational biology.

## Introduction

Ontologies are now widely used for describing and enhancing biological resources and biological data, largely following on from the success of the Gene Ontology [1]. Ontologies have been used for many purposes, from schema integration to value reconcilliation to query interfaces [2]. Ontologies have also become a cornerstone of computational biology and bioinformatics. As computationally amenable artifacts they are, themselves, a direct part of computational biology; many computational biologists are involved in their production and maintenance. Many more use ontologies to summarise their data, often by looking for over-representation [3], as the basis for drawing computational inferences about data [4], or as the basis for determining semantic similarity [5]. Even those not making direct computational use of ontologies are likely to come into contact with them, for example, when preparing annotation as part of their data release [6].

It is, therefore, of vital interest to computational biologists that ontologies for use within biomedicine are fit for purpose. One effort that aims to increase the quality of the ontologies available within biomedicine is the “OBO Foundry” [7]. The main tool that it uses for this is “an evolving set of shared principles governing ontology development”. The initial eleven principles of the OBO Foundry [8] were largely concerned with what might be termed ‘good engineering practice’ (ontologies must, for example, be openly available, with a common syntax, well documented, and used). These principles have later been joined by a further eleven [9]; these include principles such as “textual definitions will use the genus-species form”, “Use of Basic Formal Ontology” and, the somewhat quixotic, “terms […] should correspond to instances in reality”. These stem not from engineering practice, but from a perspective called *realism*.

The many different uses for ontologies that we have described are reflected in different understandings and methodologies about how and what to represent in an ontology. Over the last few years, for many uses the paradigm has moved from “a conceptualization of the application domain” toward “a description of the key entities in reality”; it is this latter approach that defines realism [10]. This approach to ontology is typified by the Basic Formal Ontology (BFO); a small upper-ontology for use within science in general and biomedical ontology building in particular [11].

There has been significant discussion regarding the possibility of representing *only* “real entities” in computational ontologies [12]. Likewise, there has been significant discussion about the philosophy surrounding realism and the role of ontology in its representation [10]. While it is argued by some that it is possible to represent *only* reality when making a domain description, there has, however, been little discussion on whether it is necessarily desirable to do so.

In this paper, we consider the implications that realism has for the choices that are open to the ontologist while they are modelling their domain of interest. In particular, we consider the implications that this has for the computational capabilities of any resultant ontology, in terms of its ability to represent scientific knowledge in a computationally amenable form, as well as the ability to perform automated inference or statistics over this knowledge. We suggest that the application of realism results in ontologies that are over-complex, awkward or limited; as such, realism falls far short of its aim of increasing the fitness-for-purpose of ontologies. This approach, therefore, is unlikely to fulfil the needs of computational biologists whom form a substantial part of both the user and developer community for bio-ontologies.

## Methods

In this paper, we take the approach of a number of worked exemplars; this is a complementary approach to an in-depth consideration of the modelling decisions for a particular area or particular ontology, which we have used previously [13], as it allows broader conclusions about the general principles of ontology development. For each section, as well as the main exemplars, a number of related examples are briefly discussed, to reinforce that the issues raised are, indeed, general.

The exemplars have been selected by several criteria. First, all the main exemplars are all taken from within biomedicine; this is also true for the majority of the related examples. Second, we have chosen exemplars that provide as wide a coverage of biology as possible. For practical reasons, third, we have chosen exemplars where the underlying science is relatively basic to much of biology and is likely to be immediately clear to the reader without significant explanation.

We have chosen exemplars requiring as little knowledge of specific ontologies as possible. We refer to only three. The first is BFO (see “What is Realism?”) which is a canonical example of a realist ontology. BFO is described as a cross-domain, upper-ontology; as a result, most terms fail the criteria given above; they are of poor biomedical relevance, and are not basic science or immediately clear. We have, therefore, also used PATO (see http://obofoundry.org/wiki/index.php/PATO:Main_Page); this defines “qualities” that we might consider attributes of other entities; so, the authors of this paper have a height, weight and shape, all of which are considered to be qualities of the authors. Finally, we use the relationship ontology [14]; this describes the relations between entities. So, for example, the height of the author *inheres_in* the author.

As discussed in this and other works [15], [16], “realism” is itself poorly defined. Where this lack of definition makes the consequences of realism hard to determine, we have taken the practical course, of showing the consequences as they play out in practice; to an extent, therefore, these three ontologies are not only exemplars for realism, but define it, as it is currently practiced. In short, for this paper, when we say “realism”, we largely mean “realism as practiced by BFO”. We do not claim, in this paper, to address all the philosophical perspectives that through time carried the name “realism”.

## Results

### What is Realism?

Building ontologies based on reality is obviously appealing to most scientists; after all the study of *reality* to determine its behaviour and laws is the goal of scientists. A brief consideration, however, shows that this notion cannot define a methodology for the building of ontologies.

Within the context of science “reality” would normally be taken to mean our experimental or observational data; but the statement that science (ontologies) should be based on experimental or observational data is a truism and, as such, has no explanatory power. The “real” in realism refers, in fact, to the belief that the categories that we can use to divide entities are, themselves, real.

This distinction stems from an old argument from philosophy; realism against conceptualism. Again, both sides of the argument agree that the world we can percieve, and as scientists, experiment on, is mind-independent. The conceptualist, however, argues that the categories that they term *concepts* are a product of social agreement. Conversely, the realist argues that these categories that they term *universals* are themselves real, that is mind independent in their own right, like the entities they describe.

This distinction may seem fairly confusing; as Russell [15] says “if I have failed to make Aristotle's theory of universals clear, that is (I maintain) because it is not clear”. In fact, there is a third possibility that is a more empirical view—that is, if categories (or other models) help in describing and predicting experimental data, then they are useful regardless of whether they are real or otherwise [17]. As an example, the Mendelian notion of segregating units of inheritance was defined and useful many years before a complete mechanistic description of their cause was available. In this context, we note that there is no commonly used term to express this form of category; most commonly, “concept” is used.

For a field with a core activity of providing definitions, there is surprisingly little agreement on the meaning of the word “ontology”; as there have been many papers on the topic, we consider just a few that reflect the distinction between these approaches. Probably the most commonly cited definition [18] describes an ontology as “a specification of a conceptualization”. This definition emphasises the formality (i.e. logical and, therefore, computationally amenable) aspect to ontology development.

This is countered with a realist definition; while the requirements from Gruber's definition—a formal specification—are necessary, realist ontologies add the requirement that “the nodes and edges correspond not to concepts but, rather, to entities in reality” [19].

What does “reality” in this context actually mean? Definitions such as “that which exists” are strangely circular leaving the question of what “exists” means. Smith [12] adds the priviso that reality is “captured in scientific laws”. Being a scientific law is not strictly enough, as some are later shown to be wrong, but a scientific law is the current best attempt at reality; this possibility does not make an ontology non-realist. For a realist ontology, the nodes are “universals”—entities in reality—rather than concepts; at least one particular must exist for every universal.

This still leaves the difficulty of applying the realist definition in practice. So most scientists will happily accept, for example, that a cell is real as it is an entity that can be observed, interacted with and manipulated. However, concepts such as “function” [13] have raised more discussion [20]; is this “real” or just a word biologists use as a point of reference? While the definition involving “entities in reality” maybe of philosophical interest, they are hard to turn into a specific assay; how to test whether a particular concept is, also, a universal. Instead of a clear assay for existence, realism offers direction about what concepts are NOT reality, rather than those that are reality. For example, and perhaps ironically given the negative practical definition of reality, a statement such as:

Dog is_a not Cat

is not held to be a statement about reality as it is a logically constructed example of subsumption (an is_a relationship); there is no real universal containing particular not Cats in existence. Likewise,

Dog is_a (Dog or Cat)

as the existence of particular Dogs and Cats does not mean that there are any particular Dog or Cats (examples modified from [12]).

This is not meant to provide a complete introduction to “realism”, but to provide a grounding for the discussion that follows; we will consider the issues raised by realism, throughout the paper. A more philosophical treatment of realism is given by Merrill [16]. It is useful to note that Gruber's [18] statement that “And it [a computational ontology] is certainly a different sense of the word than its use in philosophy.”. In this paper, we are concerned with the ontologies as computational artefacts.

To summarise, a realist approach to ontology says that the categories or universals in to which objects or particulars fall have an existence in their own right. It is these universals and *only* these universals that a realist approach says should be the nodes within an ontology. In this paper we examine whether this approach is an adequate means to provide an account for the data produced by biomedicine.

### Models that represent reality

In this section, we suggest that many universals have a range of representations. In some cases, the choice of representation may be obvious, such as length which has a natural scientific representation in SI units. In many cases, however, there is no clear set of criteria for choosing between representations. We consider the way that one quality, *colour*, could be represented ontologically.

Colour is a complex phenomenon. The colour of an object or other phenomena arises, in part, from that object and, in part, from the eye that perceives it.

A representation of the physical reality would be an account of the reflection, transmission and perception of light by an organism. Such an account of the reality of light and its perception might cover the following facts: Chlorophyll is green in reflection and red in transmission; a flower petal appears white to a human, but has UV stripes to a bee; the plant leaf and the algae appear green to humans, but have different reflection spectra because their chlorophyll co-ordinate to their Mg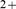 ion in different ways.

There have been a number of different attempts to represent the complexities of colour numerically, for a number of different purposes. These are models that allow us to describe colour, without having to deal with the underlying physics or reality of colour. Probably the best known of these are RGB (Red, Green, Blue) or HSV (Hue, Saturation, Value), both of which are additive colour models appropriate for describing colour on a display screen. CYMK (Cyan, Yellow, Magenta and Black) is a subtractive colour model and commonly used for printing.

Collectively these representation schemes are known as *colour models*. That none of these schemes has become predominant reflects both their different uses and the preferences of different user groups.

For the ontology builder, this leaves us with a difficult choice:

1. We bless one of the colour models, substituting the model for the underlying physics and do not describe the others.
2. We describe all of the colour models, but do not describe that they are part of a colour model.
3. We explicitly describe the reality of the physics, biology and the relationship to the different colour models, reflecting the practise of describing colour in much of science.

Currently, considering the PATO ontology, which is documented as being built according to realist principles, the first approach has been taken, using the HSV scheme. So, PATO has a term **Color Hue** (PATO:0000015) that is defined as:

*“A chromatic scalar-circular quality inhering in an object that manifests in an observer by virtue of the dominant wavelength of the visible light; may be subject to fiat divisions, typically into 7 or 8 spectra.”*

Using this model, PATO describes **red** (PATO:0000322) as:

*“A color hue with high wavelength of the long-wave end of the visible spectrum, evoked in the human observer by radiant energy with wavelengths of approximately 630 to 750 nanometers.”*

This modelling approach has a number of limitations.

- The decision to choose one colour model or the other is arbitrary. While there are reasonable justifications for the use of HSV as opposed to, for example, RGB, there is no *a priori* justification for use of an additive colour model as opposed to a subtractive model. Both are valid, for different usage; in general, reflective colour is more common in biology (e.g. pigmentation) than emitted colour (e.g. fluorescence) which would suggest that subtractive models are more generally applicable, but a full treatment requires both.
- There are no terms which can be used to express data described according to other colour models, necessitating a transformation between the different models into the officially “blessed” version during application of the ontology. These transformations may be lossy and not fully reversible.

The second approach is also possible. This would allow expression of data in multiple colour models, however:

- The ontology would tend to get rather confusing as more colour models are added; colour would have children “Hue”, “Red” and “Cyan” and seven other sibling terms.
- It is not clear which terms comprise a colour model: do values for “Hue”, “Green” and “Magenta” specify a colour?
- It is not clear whether terms that occur in the other contexts are equivalent. Is “Red as in RGB” the same or different as **Red** (PATO:0000322)? Is “Hue as in HSV” the same or different from “Hue as in HSL” (HSL is another additive colour model).

The third approach does not suffer from the limitations described. We suggest from this analysis that it is necessary, if unfortunate, for some qualities to be explicitly described with multiple representations. To avoid confusion, the universal quality, colour, would need to be explicitly described as having multiple valid models. Yet, realism argues that we should not do this, as colour is real and not a model; more over, the focus on realism means that the documentation does not describe the choices that have been made, nor refer to the relationship between **Color Hue** (PATO:0000015) and “Hue as in HSV”. In short, realism has limited our ability to represent colour.

#### Related Examples

There are many different examples of this issue; having two or more models to describe the same part of reality is common. The distance between two markers on a chromosome can be measured using (one of a number of) genetic techniques. Some qualities have a bewildering array of different measurements associated with them; Wikipedia, for example, lists 13 different measurements of concentration such as molarity or 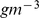.

This issue has been previously recognised. In computing science, explicitly modelling one model in another is a form of *metamodelling*. Other, non-realist, upper-ontologies such as DOLCE use the concept of Quale to describe a cognitive abstraction (such as Colour), including those over a physical quality (such as the spectral properties of reflected light) [21].

### Sequences and the Central Dogma

The central dogma of molecular biology suggests that all genetic information is encoded in the DNA of a cell, as the ordered nucleotides that comprise the DNA. RNA is transcribed from this DNA. The RNA molecule also has a defined order of nucleotides related to the DNA. Finally the RNA is translated into protein.

Consider an ontology describing these entities. First, the DNA molecule has a number of properties; as well as physical dimensions (discussed further in “The limits of consistency”), including a length expressed in metres, it consists of a number of monomeric units. So, for example, we might say a DNA molecule with a series of nucleotide residues represented as ‘GATC’ hasMonomericPart 4.

This causes a slight worry from a realist perspective; the number 4 may not be a realist universal. There are no instances of 4. In this case, the number 4 is being used to describe a part of reality, so this is allowable in a realist ontology. Alternatively, we could describe the same reality using units (traditionally base-pairs or bp). Therefore, the DNA molecule hasPolymerLength 4bp.

Accepting the use of natural numbers in this way, also means that we accept the use of sets and sequences to describe reality. One definition of 4 is a sequence. Stating that the DNA molecule represented with the sequence ‘GATC’ hasPolymerLength 4bp is equivalent, therefore, to stating that it hasSequence ‘NNNN’ where ‘N’ is any nucleotide residue.

It should be noted, however, that the usefulness of these statements stems from our *implicit* knowledge. The number 4 is a natural number, so hasMonomericPart 4.2 is not possible. If a new monomer is attached to our DNA molecule, it will now hasMonomericPart 5, because the natural numbers are additive. We understand the operation of natural numbers as part of our shared, background knowledge, and we can apply this knowledge here.

Having described that the DNA molecule represented as ‘GATC’ hasPolymerLength 4 (or hasSequence ‘NNNN’) we might wish to be more specific about the order of nucleotide residues and state hasSequence ‘GATC’, The implicit background knowledge we used previously about the natural numbers still applies here.

Next consider the process of transcription. The previous discussion about DNA likewise applies to RNA. The RNA molecule will, however, hasSequence ‘GAUL’, as RNA uses a different set of bases to DNA. Mathematically, one sequence can be determined from the other by applying a mapping; though the mapping is a human activity, not a representation of biochemical reality. To describe this, we have two options:

- Taking the realist approach, we can continue to rely on the *implicit* knowledge of the biologist, as we have previously relied on an implicit understanding of the natural numbers.
- We can be explicit about the properties of these sequences (additional to those properties shared with the naturals). We can talk about non-real world concepts such as alphabets, transformations and how these map to the real entities involved.

It should be noted that the former severely limits the ability to describe the central dogma. The transformation of DNA to RNA sequence is simple, but the transformation of RNA to protein is more complex. Again, the choice is between representing reality or representing how we practise science.

#### Related examples

The issues relating to sequences are fairly general. In computer science terms, these are abstract data types. The DNA sequence is a kind of sequence with special properties (a limited alphabet). Many of the physical quantities in science have special properties in this way. Consider:

#### Temperature

While these look like positive real numbers, temperatures are only meaningfully subtracting from each other, which gives information about heat-flow between two bodies. Other operations (addition, multiplication) which are useful for real numbers have little meaning for temperature.

#### Recombination Distance

These look like probabilities but are not, requiring a transformation to add.

There is a limitation on the ability to use abstract data types within a given ontology language; in most cases, the expressivity of the language will not allow arbitrary mathematical relations. Some languages, such as OWL, for example, provide “concrete domains”; these provide extension points within the ontology language where, for example, the special properties of temperature could be represented; other languages do not. In either case, there are limitations to these capabilities; for example, the constraint and behaviour of a concrete domain needs to be interpreted with its own semantics within a reasoner, rather than expressed explicitly within the ontology. It may make more sense in many circumstances to describe the existence of a mathematical model as discussed in “To go where science has gone before”.

### The limitations of computers

Modelling continuous properties is a common problem in ontological engineering. For example, according to statistics the western world is now facing an obesity epidemic; in short many or most of us weigh too much. Understanding, however, exactly what “too much” means is not necessarily simple; a common technique to use is body mass index (BMI)—body weight divided by square of the height, which is a continuous value. The BMI range is split into 4 categories: Obese (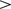
30), Overweight (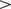
25), Normal (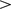
18.5) and Underweight (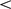
18.5). These categories represent ranges of the value of BMI.

This data simplification has many justifications. On an individual basis, the BMI is not a particularly accurate measure, so the simplification does not lose much accuracy. It is also easier to describe to patients, for whom a “BMI of 25” will be less comprehensible than being “overweight”.

Modelling some of this is straight-forward. Height and weight are modelled as properties of the individual. The BMI would therefore appear to be a property of the individual as it is a restatement of two existing properties. It would appear, therefore, that the category into which an individual falls should also be a property of the individual.

Consider the values of the property next. These categories are an abstraction over the real-world properties. Although, height as an integer value is expressed using a non-real-world entity, it is a description of a part of reality. A range, however, in the BMI does not describe part of reality in the same sense. There are no instances of BMI “Obese”. In a realist ontology, therefore, it is unclear what the relationship is between BMI Obese and the individual person.

For the statistician or computer scientist, there is an additional advantage to the simplification; four discrete groups have better computational properties than a continuous measure. Database queries become easier to write, and quicker to run. This is also true for the ontology builder; simplifying the real-world may fulfil the needs of an application for which the ontology is built, while avoiding unnecessary complexity. This is a widely used method for representing partitions of continuous values, the appropriately named *value partition* [22].

In the case of BMI there is a pre-existing social agreement toward a set of categories; however, even in the absence of such an agreement, the ontology builder might wish to represent a continuous range as a value partition to decrease the complexity of their ontology. The value partition is useful, but many of the concepts involved are not realist universals. The choice, then, is modelling “reality” and modelling a simplification that is easier to use and has better computational properties.

#### Related Examples

Splitting the two cases, there are many examples of pre-existing simplifications. From medicine, there are so many that it seems to be the norm rather than the exception: hypo- vs hyperthermic; hypo vs hypertensive; hypo- vs hyperglycemic. In many cases, these ranges have standard interpretations akin to the BMI.

There are likewise a number of constructions or design patterns that reduce complexity, extend the effective capabilities of the language or simply provide standard solutions to common problems [23].

### To go where science has gone before

Many experiments in biomedicine require the measurement of some physical property of a biological system. Take, for example, the measurement of heart rate; in standard practice, this is measured in beats per minute, and is calculated simply by counting beats (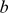) over a time period (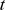) and dividing one by the other (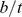). However, what time period is appropriate? We might choose 60s, but this raises the question, what is the meaning of heart rate over shorter periods?

Fortunately, there is a standard solution to this problem, which is to define heart rate using differential calculus; so heart rate becomes 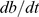.

The derivative, 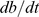, presents some problems from a realist perspective. As noted previously (see “Sequences and the Central Dogma”), it is possible to associate real numbers with entities; however, 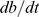 is 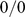. It is not clear whether this quantity is a universal; it is certainly the case that the expression 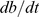 is not a universal, yet such values and calculus itself is apowerful tool within science and not using it within ontological models is a severe restriction. We can describe this ontologically in three ways:

- We can model the real world entities involved – beats, time and describe nothing else.
- We can describe rate in mathematical terms. In this case, we are defining the heart rate as a mathematical abstraction.
- We can model the heart rate as a real world entity, 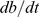 as a mathematical entity and explicitly state that 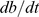 is a model of heart rate.

These different solutions present different advantages. The first is consistent with realism. The second is consistent with the most common definition used within science. The third is consistent with both but it is unclear when to use which term (for example, is 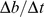 an approximation of 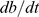, a quantification of the real world quality or both)?

In most cases for the description of science, the second option makes most sense; conflating the mathematical model with the real entity enables us to use the advantages of two different modelling techniques without introducing the confusion of the third option.

#### Related Examples

There are many related examples from mechanics, electromagnetics or chemistry; as with value partitions in medicine, so many that they appear to be the norm. All of these subject areas have direct relevance to biology and, perhaps even more so, to the equipment used in the practice of biology.

Mechanical examples would include velocity (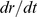) and acceleration (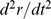). Electromagnetics would include current (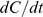) and capacitance (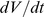). Chemistry examples would include rate constants and pH. In biology, population biology, systems biology and neurosciences make wide use of mathematical models. The lack of a link in realist ontologies to these mathematical models is not free from consequences (described further in “Discussion”).

The more general issue comes not from relating to differential calculus, but relating to pre-existing non-ontological techniques. For example, taxonomy in the linnean sense. There have been many discussions about whether species and high taxons are reflective of reality; it is certainly the case that a number of higher taxons do not reflect phylogeny [24]. Given that it is of uncertain status, should we represent taxonomy as a quality of an organism, an independent conceptualisation of the biologists or both?

### The limits of consistency

Physical biological entities such as cells and organisms have an extent in the real world. This paper's first author, for example, has a height of around 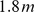; a similar value cannot be applied meaningfully to the electronic version of this document, although it may apply to the paper that it may be printed on.

There are a number of different, well-understood mechanisms for representing physical space. We can use a dimensional or cartesian model, with three perpendicular lines with a linear scale. We can use a polar model, expressing extent using angles and a single distance. Modern physics has told us, however, that all of these are limited models of reality; physics generally uses a four dimensional Minkowskian spacetime model; here the axes are not linear; motion of the observer down one will change values down the others. Alternatively, at a quantum level, length is a probability distribution.

For the ontology builder, this leaves a difficult choice and the same choice discussed previously in “Models that represent reality”: Represent the reality physicists relate; bless one, ignore the rest; describe their components but not their models; explicitly describe them.

If the ontology builder is to be consistent, then, they should make the same choice in both cases; if we describe colour models, we should explicitly describe Minkowskian spacetime, quantuum probability distributions, cartesian and polar systems.

There are, however, two important differences to colour models. First, there is a strong social bias toward cartesian systems. Secondly, within the scope of biology and the life sciences, four dimensional spacetime or quantuum models confuse rather than simplify; the relativistic corrections produce such small differences that they are statistically meaningless; similarly, describing a leg as a probability distribution adds little other than complexity.

This leaves the ontology builder with two options:

1. We can build an ontology with a consistent relationship to reality. So, having decided to explicitly represent colour models, this suggests that we should also explicitly model 3D space, 4D spacetime and the various co-ordinate systems that are used to describe these.
2. We build an ontology with an inconsistent relationship to reality. So, we might be explicit about colour models, but arbitrarily bless 3 dimensional space, using cartesian co-ordinates.

The compromise here is very straight-forward. The first solution retains its consistency to reality, the second is consistent with usability and usage; for biomedicine, a 3D cartesian co-ordinate system plus time is likely to be enough for the foreseeable future and makes life easier in the meantime.

The Newtonian view of the world is the best model in this case: it is good enough. When building an ontology for biomedicine, it makes most sense to use this view as it will produce the results required. If, in the future, biomedicine advances so that relativistic or quantuum representations are necessary, then current ontologies will need refactoring; even then, this future cost is likely to be offset by gains in the present.

#### Related examples

In the choice of units for measurement for scientific purposes, SI units are to be preferred. It should be noted, here, that there is a domain dependency; for an engineering ontology, the use of American imperial units would be inevitable.

For most of biology it is unnecessary to distinguish between the length of the calendar year and the astronomical year—the latter changing with respect to variability in the motion of the earth. There are occasions when this distinction may be important for data integration in bioinformatics as leap years and leap seconds show.

For an ecologist counting the number of trees in a sampling square 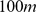 by 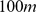, they will take the area as 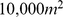; The surface is, however, neither smooth nor a Euclidean plane, so this area is wrong in reality. For much of ecology, this distinction will not matter. Again, there is a domain dependency here; whale or bird biologists interested in migration patterns may well care about the curvature of the earth.

## Discussion

Realism has been held up as a methodology for “good” ontological modelling, and the production of more tightly defined and consistent ontologies. In this paper, we have discussed five different cases, with biological examples, that we might wish to model ontologically; for each, we have presented different models, describing the same underlying science. In each case, a realist solution is possible, but places either limitations or awkwardness on the models produced.

Building an ontology with a consistent relationship to reality may help to enable interoperability [7] under some circumstances. If, however, it disallows modifications for computability (see “The limitations of computers”), or requires arbitrary blessing for one form of specification over another (see “Models that represent reality”) it may have the opposite effect.

Nor are the issues discussed in this paper free from consequences. In “To go where science has gone before”, we discussed interoperability with existing scientific models. Mathematics and physics have produced complex, refined and expressive notation systems, representing a deep understanding of how numbers and the physical world work. These are, however, not being used in current ontologies and this results in a lack of precision, errors and omissions:

### Lack of Precision

The PATO term **speed** (PATO:0000008) which is defined as :

*“A physical quality inhering in a bearer by virtue of the bearer's rate of change of position”*

with a synonym of velocity; from this definition, we cannot distinguish the vector and scalar quantities of velocity and speed; indeed, it is not clear which of these two **speed** (PATO:0000008) is. Meanwhile **acceleration** (PATO:0001028) is defined as:

*“… the rate of change of the bearer's velocity in either speed or direction”*

which is implicitly a vector quantity, and contradicts the statement that speed and velocity are synonyms. The mathematical definitions (velocity as 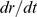, speed 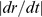, acceleration 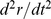) are precise, concise and accurate.

#### Errors

Similarly, **length** (PATO:0000122) is defined as a quality; qualities have to inhere in Independent Continuants; as a Spatial Region is a child of Continuant this means that Spatial Regions cannot bear lengths. In short, in current versions of BFO, there is no intuitive way of modelling the length of a region in space.

#### Omissions

BFO is mass-centric; it is currently unclear where many physical entities exist, examples including energy, waves (through a medium) or EM radiation. Likewise, it lacks a natural position for numbers (that have no particulars), patterns and distributions. Yet, these entities are key to a physical description of the world.

To our mind, these are indicative of some of the most serious flaws of realism-based ontology building. It makes little sense to replicate the models of physics using English instead of a more precise mathematical notation. If BFO had been built using direct links to a grounded physical model of the world, it seems likely that these problems would not have arisen.

We have discussed a number of concrete examples where building an ontology by considering realist concerns has detrimental consequences for the model. We believe that the real world entities and the relationships between them is only one consideration among many: simplicity, usability, fitness for purpose are equally important.

Taken to its most extreme form realism, it seems to these authors, would produce models unsuitable for use within science. There is a choice between a correct account of reality that does not allow the data of science to be adequately described and a description of reality that takes in to account how science is performed. Fortunately, most “realist” ontologies are not really so: PATOs representation of HSV for modelling colour is not a bad decision; it represents a straight-forward, pragmatic approach to ontology building, where the representation has been chosen on the basis of a use case, not the entities as they exist in reality. Similarly BFO uses a 3D plus time model of reality; it suggests that length are properties of the entity alone, without reference to the observer. This is not a true reflection of reality, but one which is a good enough approximation for use within the biomedical sciences; in short, usability and simplicity have been considered to be more important in the modelling process than the relationship of the model to reality. In accepting these compromises, BFO has placed itself squarely as a computational rather than philosophical ontology.

Despite these concerns, realism has made a contribution to the field of biomedical ontology engineering. By emphasising the importance of real-world entities and by encouraging a more specific interpretation than the generalisation of a “conceptualisation”, realism helps to avoid the introduction of unnecessary layers of abstraction. A consideration of the entities in reality may be a part of an ontology engineering process; ontology builders should have careful and considered reasons for diverting from modelling in this way and that ontologies should explicitly describe through annotations the terms that do or may divert from this view. Ontology builders should, however, be free to make this decision; the acceptance of compromise with respect to reality will result in simpler and more effective knowledge artefacts.

Johansson [10] when discussing realism asks the rhetorical question: “would you like to be treated for a physiological illness by a *(non-realist)* physician who is not sure that there are human bodies?” – (our emphasis). As scientists, our reply would be if their survival and success statistics were the best, we would not care whether they were a realist, a non-realist or a robot which admitted of no philosophical position at all; also, using a doctor who was strictly realist and thus cut off from much of the practise of science (such as determining heart rate) would disturb many patients. As bioinformaticians, we build ontologies to provide a descriptive and predictive model of the wealth of experimental data that is now available. In biology, the job of an ontologist is to describe data such that it can be analysed. Naturally this entails a description of entities in reality; it also, however, entails a description of science, and it entails compromise; we overlook this to our peril. The last 200 years of science shows the success and strength of this position; it is on this groundwork that we should build for the future.
